# Identifying Ciliary Proteins in Mammalian Retinas using a Gentle Extraction Method

**DOI:** 10.17912/micropub.biology.001218

**Published:** 2024-06-13

**Authors:** Adeline S. Fredrick, Erin R. Claussen, Samantha N. Fischer, Samson Balasanyants, Akshaya Rajaraman, Andi C. Rosner, Kevin Drew

**Affiliations:** 1 Biological Sciences, University of Illinois at Chicago, Chicago, Illinois, United States

## Abstract

Mutations in retinal primary cilia are responsible for human blindness but the mechanisms are not fully understood (Wheway et al., 2014). Characterizing the proteome of an organelle such as cilia, is a fruitful way to understand its function but methods often require considerable sample quantities. Here we develop a method to isolate the primary cilia of photoreceptor cells from bovine retinas. Through LC/MS/MS proteomics analysis we identify proteins enriched for cilia function including ciliopathy disease. This study shows our method can be used to isolate retinal primary cilia to obtain sufficient quantities of native protein samples.

**Figure 1. Isolation and proteomic analysis of primary cilia from bovine retinas:**
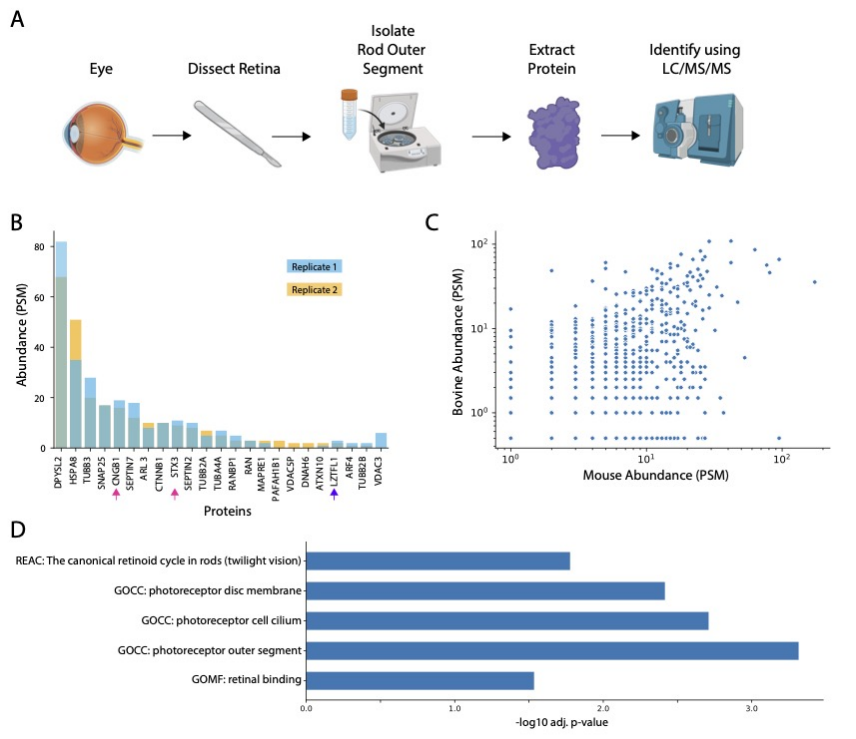
( **A** ) A schematic of the methods used to conduct this experiment. The bovine eye was dissected using scalpels and forceps. Next, the rod outer segment of the primary cilia was separated from the retinal tissue using centrifugation. The proteins were extracted and then identified using mass spectrometry (LC/MS/MS). ( **B** ) From our MS analysis we identified ~1200 bovine proteins; we cross referenced the proteins in our sample with the Syscilia database gold standard and found 23 proteins associated with cilia in our sample. The graph shows the abundance of 23 ciliary proteins within replicate samples. Pink arrow=RP associated proteins and purple arrow=Bardet-Biedl Syndrome associated protein. ( **C** ) Comparison of our MS bovine protein abundance data compared to proteins identified in mouse retinas (Liu et al., 2007). ( **D** ) Annotation enrichment analysis of the 40 most abundant proteins in our sample and select significant annotations. Proteins were analyzed by filtering the top 40 proteins by total PSMs across both replicates. (REAC=Reactome, GOCC=Gene Ontology Cellular Component, GOMF=Gene Ontology Molecular Function, PSM = peptide spectral match) (Images in Figure 1A were created with BioRender).

## Description

Retinitis pigmentosa (RP) is a rare eye disease that results in vision loss for 1 in 4000 in the United States and has no cure (O’Neal & Luther, 2024) . A common cause of RP is disruptions in the primary cilia of the retina, the tissue responsible for conducting visuals from the eye to the brain through neural connections. To better understand the molecular causes of RP, studies have focused on identifying proteins in retinal primary cilia (Liu et al., 2007) but have been limited in detailing the full protein interaction network. High throughput proteomics experiments that characterize protein complexes and protein interactions often require a large amount of biological material (Drew et al., 2020; Mallam et al., 2019) . Large mammals such as bovine ( *Bos taurus* ) are an attractive model to fill this need. In particular, the bovine eye is comparable in size to the human eye and ~10x larger than the mouse eye (Kralik et al., 2023) . Moreover, bovine eyes are diurnal (same as humans) and highly visual as opposed to mice being nocturnal, making bovine retinas a better model for human vision (Kralik et al., 2023) . The goal of this research is to develop a gentle extraction method to isolate native complexes from primary cilia of bovine retinas. Here we describe our method to obtain retinal primary cilia sample and demonstrate the identification of proteins associated with retinal primary cilia.

Figure 1A describes our experimental workflow . We first acquired and dissected 10 bovine eyes to extract retinal tissue. We then isolated the modified primary cilia of the retina known as rod outer segments through centrifugation. We measured the final protein concentration of our sample to be 0.5mg/ml and resulted in an estimated ~1 mg total protein. Finally, we prepared the sample for bottom-up proteomics for protein identification using LC/MS/MS. Two replicate samples were sent for proteomic analysis where ~1,200 proteins were identified (see ExtendedData Table 1). To analyze these data, we compared our identified proteins with the Syscilia database, a collection of human ciliary proteins (Vasquez et al., 2021) . Comparing our data with the Syscilia database, 23 proteins were found that our bovine sample had in common with human ciliary associated proteins ( Figure 1B ). Of these 23 proteins, three of them were directly linked to human ciliopathies. Specifically, CNGB1 and STX3 are associated with RP which affects how the retina responds to light. CNGB1 is a coactivator for transcription, cell adhesion, and neurogenesis. STX3 is important for docking synaptic vesicles and for membrane fusion. We also identify LZTFL1 which is linked to Bardet-Biedl Syndrome (BBS), a ciliopathy that affects rod and cone development in the retina, renal formation, mental development, and obesity (Marion et al., 2012) . To demonstrate our identification of the ciliary proteome in an orthogonal analysis, we used GO enrichment to identify enriched functional annotations within our identified proteins. Specifically, we searched for annotations that were enriched in the 40 most abundant proteins and found terms associated with the photoreceptor outer segment (GO:0001750, p _adj_ 4.836×10 ^-4^ ), photoreceptor cell cilium (GO:0097733, p _adj_ 1.952×10 ^-3^ ) and other related terms ( Figure 1D ). Collectively, this is evidence that the proteins identified through our retinal cilia extraction method from bovine retinas are associated with human ciliopathy diseases and generally, retinal ciliary proteins.

We finally compared the proteins identified from our bovine retinal sample to a sample obtained in a similar manner from mouse retinas (Liu et al., 2007) ( Figure 1C ). The Spearman correlation coefficient between the two datasets was 0.394 showing that our data is partially correlated with the mouse retina data. We do however see overlap among the most abundant proteins including Pkm, Map1b, Sptbn1(Fodrin), and Atp1a3, which are known to localize and function in neurons. In total, we identify 1625 proteins (PSM > 1) compared to 1058 identified proteins by Liu et al. (PSM > 1). Taken together, this suggests that the high abundant proteins identified by our gentle extraction method are in agreement with previous studies and our study extends the number of identified proteins in the retinal primary cilium overall. In sum, this research is important to understand the proteomics of primary cilia, to procure a gentle extraction method to understand what constitutes these cilia, and to be able to research diseases associated with proteins within the cilia.

## Methods

We followed the protocols outlined by Panfoli and colleagues (Panfoli et al., 2022) for the dissection of retinas from 10 bovine eyes. In a dark room, we cut around the lens of the eyes with a scalpel and removed the vitreous humor. Once the eyes were hollowed, we incubated them in a mammalian ringer solution for 15 minutes on an orbital shaker at low speed. Using scissors, we severed the optical nerve from the back of the retina. We used forceps to remove retinas and placed them in a 50ml conical tube. Once retinas were isolated, we followed the protocols from Liu and colleagues (Liu et al., 2007) to isolate rod outer segments. We applied the sample to a 50% sucrose cushion in Buffer A and centrifuged for 20 minutes at 13,000 g. We recovered the interphase, diluted in 1:1 buffer A, and centrifuged for 20 minutes at 13,000g. We then incubated the pellet in buffer B on ice for 1 hour, occasionally pipetting up and down. To determine protein concentration in the sample, we used the detergent-compatible BioRad DC protein assay kit. The sample was then processed twice as two separate technical replicates for bottom-up proteomics. To prepare samples for MS analysis, samples were diluted to a final concentration of 5% TFE by adding 880 µl of digestion buffer. Trypsin was added to a final concentration of 1:25-1:100 enzyme:protein (2µg trypsin for 1-2 mg/ml). Samples were desalted using C18 spin tips. Peptides were analyzed using a ThermoScientific Acclaim PepMap 100 C18 HPLC Column (75-minute gradient) followed by nano electrospray-ionization and tandem mass spectrometry on a Thermo Q Exactive HF. Data-dependent acquisition was activated, with parent ion (MS1) scans collected at high resolution. Ions with charge 1 were selected for collision-induced dissociation fragmentation spectrum acquisition (MS2) (HCD, collisionEnergy=28.0). MS was acquired in the UIC Proteomics Core Facility. MSBlender was used for mass spectra lookup for protein identification (Kwon et al., 2011) using Uniprot bovine unreviewed proteome (UP000009136_9913). For annotation enrichment analysis we used G Profiler (Kolberg et al., 2023) . Additional description of method details can be found in Extended Data Text: Identifying Ciliary Proteins. In addition, we performed label-free quantification (LFQ) using Fragpipe (version 21.1) built-in workflow “LFQ-MBR” with default settings, using the same database as before. The mzML files were converted from raw data using Proteowizard (version 3.0.20287) (Kessner et al., 2008) MSConvert. MSFragger (version 4.0) was performed using default closed search parameters with mass calibration and parameter optimization (Kong et al., 2017) . Peptide-spectral match validation was performed using Percolator (Käll et al., 2007) . Protein inference and FDR filtering were performed with ProteinProphet (Nesvizhskii et al., 2003) from the Philosopher (v. 5.1.0) toolkit (da Veiga Leprevost et al., 2020) . These results can be found in ExtendedData Table 2.

**Data Availability:**

The mass spectrometry proteomics data have been deposited to the ProteomeXchange Consortium via the PRIDE (Perez-Riverol et al., 2021) partner repository with the dataset identifier PXD051952.

## Reagents

**Table:** 

Mammalian Ringer Solution (1L)	Mix the following into 500mL MQ water, add solution to 1000mL graduated cylinder and bring to volume. pH to 6.9: 157 mL 1M NaCl, 0.5 mL 1M KCl, 3.5 mL 200mM Na _2_ HPO4, 4 mL 200mM NaH _2_ PO _4_ , 0.05 mL 1M MgCl _2_ , 0.05mL 1M CaCl _2_ , 2mM glucose (0.36g), cOmplete, EDTA-free Protease Inhibitor Cocktail, Roche
Buffer A (1L)	10mM Pipes, 5mM MgCl _2_ , cOmplete, EDTA-free Protease Inhibitor Cocktail, Roche, pH 7
Buffer B (1L)	10 mM Pipes, pH 7.0, 5 mm MgCl _2_ , Triton X-100, 1 mm DTT 1%, EDTA-free Protease Inhibitor Cocktail, Roche
Digestion Buffer	50mM Tris-HCl 2mM CaCl2 pH 8
Bovine Eyes	Sierra For Medical Science, Whittier, CA

## Extended Data

Description: ExtendedData Table 1 Bovine Retina Mass Spec. Resource Type: Dataset. DOI: 10.22002/wj77d-mpj78

Description: ExtendedData Table 2 Bovine Retina LFQ Mass Spec. Resource Type: Dataset. DOI: 10.22002/5nnjr-9fy77

Description: Identifying Ciliary Proteins. Resource Type: Text. DOI: 10.22002/qjwch-e0t24
